# Factors beyond Workplace Matter: The Effect of Family Support and Religious Attendance on Sustaining Well-Being of High-Technology Employees

**DOI:** 10.3390/healthcare9050602

**Published:** 2021-05-18

**Authors:** Ing-Chung Huang, Pey-Lan Du, Long-Sheng Lin, Tsai-Fei Lin, Shu-Chun Kuo

**Affiliations:** 1 Department of Asia-Pacific Industrial and Business Management, National University of Kaohsiung, Kaohsiung 811726, Taiwan; ichuang@nuk.edu.tw (I.-C.H.); fatimakuo0901@gmail.com (S.-C.K.); 2 Department of Sport & Leisure, National Quemoy University, Jinning Township 892009, Kinmen County, Taiwan; peylandu@gmail.com; 3 Department of Business Administration, Tainan University of Technology, Tainan 710302, Taiwan; 4 Institute of Human Resource Management, National Sun Yat-Sen University, Kaohsiung 80424, Taiwan; tsaifei.lin@gmail.com

**Keywords:** family support, work engagement, subjective well-being, religious attendance, JD-R model, moderated mediation

## Abstract

Background: Apart from the workplace, drawing support from family and religion is critical to maintaining the well-being of high-technology employees. Relying on the job demands-resources model and the positive affective spillover effect, the aim of this study was to investigate the mediated relationship of family support, work engagement and subjective well-being, and the moderating effect of religious attendance on the mediated relationship. Methods: A cross-sectional research design was adopted. Mediation and moderated mediation were tested using the PROCESS macro v3.5 for the SPSS supplement. Purposive sampling was used for the distribution of questionnaires to high-technology employees in Taiwan. Results: Results from the data of 603 high-technology employees indicated that family support, work engagement, and subjective well-being exhibited a significant mediated relationship, and the mediated relationship was stronger among individuals with religious attendance experience. Conclusions: This study emphasizes the driving effect of family support on high-technology employee well-being and the moderating effect of religious attendance as a situational strength. We recommend closely attending to employee well-being because doing so is conducive to both the personal quality of life of employees and the sustainable development of organizations.

## 1. Introduction

Employee well-being can elicit creativity [1] and innovative behavior [2]. Moreover, employees perform better when they are healthy and happy [3,4]. Therefore, raising employee well-being is a critical topic in organizational development [5]. Research has indicated that social support is connected with work-related well-being [6], such as work engagement [7,8], and it is also connected with subjective well-being in personal life [9,10]. Through the positive affective spillover effect, employee positive affects derived from perceived work engagement in the workplace extend to their personal life, which enables them to experience subjective well-being [11,12]. This has prompted researchers to explore the mediated relationship of social support in the workplace, work engagement and subjective well-being [12]. For example, the positive affects generated in the workplace from supervisor support can be transferred to employee personal life.

According to the job demands-resources model (JD-R), job resources are related to well-being and can therefore help employees cope with job demands [13]. Job demands and resources are the two factors that may aggravate or ameliorate job stress, influencing work performance and employees’ physical and mental health through different mechanisms [14]. Job demands trigger the health impairment process, which reduces the physical and psychological resources of employees and impairs their physical and mental health. Job and personal resources increase job motivation in employees through the motivation process [14]. Therefore, having appropriate job resources enables employees to be committed to their work and achieve work performance [15]. Meanwhile, job resources mitigate the undesirable effects of job demands on employees [16]. For the high-technology industry, the rapid technological advances drive organizations to constantly search for product innovation [17,18]. This forces employees to work overtime [19] and spend most of their time at work [20]; the ever-increasing workload and responsibilities thus impose tremendous stress on employees [21]. Based on the JD-R model, the sources of job resources are related to primary life territories; in the case of high-technology employees, work and family are considered their most essential life territories [22]. Hence, the ability to draw resources from the workplace and from family is a key factor to employee well-being. However, among employees in high-technology industries, support from colleagues may have a different effect than support from family members.

In job-related matters, however, high-technology employees tend to work independently, because they are capable of accomplishing work alone and may have key knowledge of the industry [19]. Receiving assistance from colleagues may be interpreted as a sign of incompetence, and such a fear often acts as a deterrent against seeking help from colleagues [23,24]. Moreover, mutual support between colleagues can create mental burdens for employees who have received such support, because they have to repay or reciprocate favors rendered, especially when they feel obligated to return [25], and this is detrimental to positive affects in the workplace [23]. In addition, a mutually supportive ambient between colleagues can result in social loafing, which is harmful to work engagement because it lowers work motivation [26]. Therefore, receiving colleague support may act negatively on employee motivation for work and positive affects in the workplace [23]. By contrast, resources from family support can be drawn without much concern, enabling them to become more devoted to work, build positive effects, and achieve greater performance in the workplace [27]. Accordingly, obtaining family support may become the critical factor affecting the well-being of high-technology employees. The relationship between family support, work engagement, and subjective well-being merit further investigation.

Aside from job resources in the physical world, high-technology employees facing constant work-related stress can turn to the spiritual world (i.e., religion as a source of resources) [28] for work predicaments [29] and for success at work [30]. Associating with people sharing the same faith in religious ceremonies or activities is a situation that enables one to trust others [31], to relax and be relieved from stress by being temporarily away from troubles [32], and to experience well-being through interactions with people sharing the same values [31]. Both in Eastern and Western culture traditions, adherents and believers interact by attending religious activities and provide emotional support to one another that promotes well-being [28,33]. Therefore, religious activities are a type of activity in which high-technology employees may be willing to participate in their free time. This is particularly true considering that individuals more experienced in religious attendance have more access to learning of religious doctrines [33,34] and are more inclined to accept family support [35], both of which are beneficial to work engagement. Thus, religious attendance can be considered a situational strength [36] that moderates the effect of family support on work engagement and may influence subjective well-being.

Accordingly, apart from the workplace, family and religion are also primary territories in a person’s life [22]. Therefore, drawing support from family and religion is critical to maintaining employee well-being. Most studies on moderators to the relationship between social support and work engagement have focused on situational constraints such as job demand [37,38]. However, potential moderators from situational strengths [36] also warrant discussion.

The present study examined the mediated relationship of family support, work engagement and subjective well-being, in addition to exploring the moderating effect of religious attendance, particularly for employees with a high level of job demand. Employees in the high-technology industry typically experience stress from high job demand, and such stress is deemed to have a negative effect on employee health and work-related attitudes [19,20,39].

In this study, we applied the JD-R model and the positive affective spillover effect to explain moderating and mediating effects. The JD-R model demonstrates the importance of job resources for increasing well-being in a stressful situation. That is, the work engagement-related positive effects that an employee develops because of job resources such as religion or family support can spill over to personal life, thus providing subjective well-being. Accordingly, we hypothesized that for individuals with religious attendance experience, such experience would strengthen the mediating effect between family support, work engagement, and subjective well-being. Therefore, the aim of this study was to investigate the mediated relationship of family support, work engagement and subjective well-being, and the moderating effect that religious attendance has on the mediated relationship. The hypothesis development is presented below.

### 1.1. Family Support, Work Engagement, and Subjective Well-Being

#### 1.1.1. Family Support

Family support is critical to employees because it motivates innovative behaviors and mitigates job stress. Especially in the creative thinking stage, employees require emotional support from their families [40]. Family support provides psychological resources for employees to buffer job stress and cope with onerous job demands [41].

#### 1.1.2. Work Engagement

Work engagement denotes a positive psychological state at work and usually reflects three characteristics of employee work-related well-being: vigor, dedication, and absorption [42,43]. Work engagement is a dynamic state that improves after employees augment their personal resources [44]. When employees engage with their work, they are motivated to utilize their personal resources toward attaining job goals [42].

#### 1.1.3. Subjective Well-Being

Subjective well-being includes cognitive and affective components [45] and refers to personal subjective evaluations to their lives and emotions [46]. Individuals assess their own well-being according to how they generally feel toward their life experiences and emotionally respond to their life events [47].

#### 1.1.4. The Mediation Relationship

Employees with a high level of work engagement are energetic, enthusiastic, and immersed in work-related positive affects [6,48]. Family support is a specific type of social support that helps individuals achieve work objectives and develop positive affects while working [27,49]. Because positive affects generated at home from family support also improve the well-being at work and work performance [48,50], according to the JD-R model, employee work engagement can be enhanced through the motivational process if family support is gained [15].

In a broader sense, subjective well-being encompasses life satisfaction, happiness [45,51], and joyfulness [52]. Happiness and subjective well-being share similar characteristics such as subjectivity, positive affectivity and experience, and a comprehensive evaluation of personal life [46,51]. Therefore, subjective well-being and happiness can be applied interchangeably in research [53]. Interacting with family usually enhances happiness and life satisfaction [54]; for individuals who face a predicament, family support plays a critical role in making life happier and more satisfactory [9]. Family members can provide valuable support in the arena at work and at home, bringing them the feeling of happiness and meaningfulness, which leads to their overall subjective well-being [41]. Therefore, employees who have family support can experience high subjective well-being.

Through the affective spillover effect, individuals can extend well-being at work to personal life. By doing so, they can enhance work engagement and subjective well-being [11,12]. Furthermore, employees with high work engagement are usually able to feel happiness from it [38]; they can even feel similar positive affects in personal life. According to the JD-R model, job resources promote employee work engagement and subjective well-being [55]. Work engagement is possibly an effective response to the encouragement or support received from family, and such positive work-related experience and emotional response can, in turn, lead to overall subjective well-being. Thus, individuals who perceive work engagement attributable to family support will experience subjective well-being. Accordingly, we proposed the following hypothesis:

**Hypothesis 1** **(H1).** *Work engagement mediates the relationship between family support and subjective well-being.*

### 1.2. Moderating Effect of Religious Attendance

Research has confirmed that religion is related to subjective well-being [31,33,56]. From the perspective of social integration [31], religion can serve as a social resource [34]. Religious activities provide a trustful environment in which people can interact, socialize, and provide mutual affective support, thus enhancing their sense of well-being [31,33,34,57]. Individuals who frequently attend religious activities develop a strong sense of belonging, because they share the same values as their peers [32]. The psychological resources they draw from socializing with people of the same faith also help them cope with stressful events, including stress from work [32]. From the perspective of social control, fundamental rules regarding family relationships propounded by a religion have unofficial binding power [31], but religious beliefs with deep-rooted moral values can guide people toward a positive family relationship [32].

A family often attends religious activities together, and sharing the same faith also increases the opportunities of family members to exchange ideas and provide mutual affective support [57,58]. This, in turn, strengthens a family’s mutual trust and sense of belonging, which contributes to their sense of well-being [59]. Additionally, numerous religious doctrines also encourage understanding of family responsibilities [58], which has the effect of reducing family problems, increasing family support [35], and facilitating work engagement through successful completion of work objectives. Therefore, religious attendance can be considered a situational strength [36] that enhances the positive relationship between family support and work engagement. Increasing the chance of experiencing work engagement can enhance positive experiences and affective responses, thus contributing to the development of subjective well-being.

**Figure 1:**
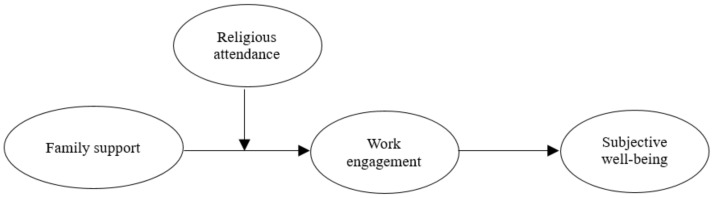
The proposed research framework.

Accordingly, we proposed the following hypothesis and research framework (Figure 1):

**Hypothesis 2** **(H2).** *Religious attendance moderates the strength of the mediated relationship between family support and subjective well-being through work engagement such that the mediated relationship is stronger among individuals with experience in religious attendance.*

## 2. Materials and Methods

### 2.1. Participants and Procedures

A self-reported questionnaire survey was conducted, where the researchers explained to the participants that the survey was conducted solely for academic research and the questionnaire responses were anonymous. A cross-sectional research design was adopted, and the researchers collected all the questionnaire data from participants at a specific point in time. This study endeavored to obtain representative samples by using purposive sampling. Five typical high-technology organizations were approached. Managers of these organizations were contacted by researchers who explained the research purpose and procedures. We collected data from high-technology firms that provide semiconductor manufacturing services, such as assembly and testing, located in an export-processing zone in Taiwan. Survey packages were distributed to 637 high-technology employees, and 603 valid questionnaires were returned, with an overall response rate of 94.7%. Verbal informed consent was obtained from participants at the beginning of responding to the questionnaire. Among the respondents, 43% were male, and 57% were female; 52.6% had at least a bachelor’s degree; and 88.6% worked as nonsupervisory employees. The average age was 33 years (SD = 9.5), and the average tenure at their organization was 6.8 years (SD = 6.9); 27.2% of respondents participated in religious activities, although they must spend most of their time at work.

### 2.2. Measurements

Participants were asked to recall their experiences from the past month in the questionnaire. Family support and work engagement were measured using 5-point scales (1 = *strongly disagree* to 5 = *strongly agree*), and subjective well-being was measured using a 4-point scale (1 = *strongly negative* to 4 = *strongly positive*).

*Family support* was measured with a four-item Chinese edition scale that Wong, Lin, and Liu [60] adapted from the family support inventory scale developed by King et al. [61] to assess individual perceptions of work-related support from family members. An example is “When something at work is bothering me, members of my family show that they understand how I’m feeling.” The Cronbach’s alpha was 0.87.

*Work engagement* was measured with a 17-item Chinese edition scale that Lien [62] adopted from the employee version of the engagement scale developed by Schaufeli et al. [6]. Example items include “At my job, I feel strong and vigorous,” “I am enthusiastic about my job,” and “I feel happy when I am working intensely.” The Cronbach’s alpha was 0.95.

*Subjective well-being* was measured using a short 10-item Chinese version of the Chinese Happiness Inventory [53,63,64]. Each item includes four statements. Respondents were asked to select the statement that most accurately represents their subjective sense of well-being. An example is “I (*never*, *seldom*, *often*, or *always*) experience joy and elation.” The Cronbach’s alpha was 0.91.

*Religious attendance* was assessed as a dichotomous variable. This variable was dichotomized into 0 (*no*) or 1 (*yes*). To prevent errors due to misreporting, forgetting, or unwillingness to provide attendance frequency for a specific period [65,66], respondents needed only to express whether they still participate in religious activities although they spend most of their time at work.

### 2.3. Data Analysis

Before hypothesis testing, missing values analysis was conducted. Variable data were missing at rates of 0.3% to 3.8%. Missing data were replaced through median imputation [67]. The final data set comprised that of 603 participants. Subsequently, descriptive statistical analysis and correlation analysis were performed. Mediation and moderated mediation were tested using the PROCESS macro v3.5 for SPSS supplement (Models 4 and 7) (IBM Corp., Armonk, NY, USA) [68] with 5000 bootstrap resamples. Model 4 was applied for mediation analysis, whereas Model 7 was employed for moderated mediation analysis. The results were used to calculate the regression coefficients for the mediation relationship and indirect effect, and the regression coefficients for the moderated mediation relationship, conditional indirect effects, and index of moderated mediation. Demographic variables including age, gender, and tenure were also used as control variables [27,48,69].

### 2.4. Assessment of Common Method Variance

The present study collected data from employees concurrently. Single-source bias might have affected the relationships between variables [70]. To reduce the bias resulting from the efforts to provide socially desirable responses [71], all data were anonymized, and respondents were assured of their confidentiality. Furthermore, to assess common method variance, we performed Harman’s one-factor test [72]. All measurement items were loaded into exploratory factor analysis. Four factors were extracted from the unrotated factor structure. The first general factor accounted for only 40.04% of the variance [73]. Therefore, common method variance was not a considerable concern in this study.

## 3. Results

### 3.1. Descriptive Statistics and Correlations

The descriptive statistics and correlations between variables are presented in Table 1. Family support was significantly correlated with work engagement and subjective well-being, and work engagement was significantly correlated with subjective well-being.

### 3.2. Mediation Hypothesis Testing

Table 2 reveals the results of the mediation analysis; the indirect effect of family support on subjective well-being through work engagement (B = 0.144, bootstrapping CI = 0.101, 0.187) was significant. The results thus support Hypothesis 1.

### 3.3. Moderated Mediation Hypothesis Testing

**Figure 2:**
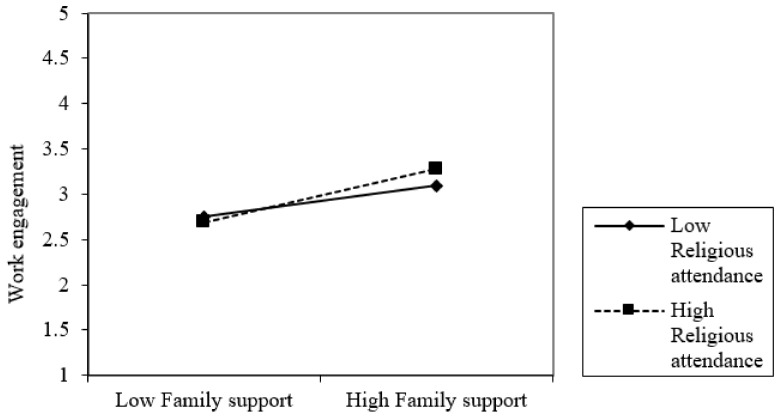
Moderated effect of religious attendance on the relationship between family support and work engagement.

Figure 2 reveals that the effect of family support on work engagement was greater among employees who experienced religious attendance. The index of moderated mediation (B = 0.083, bootstrapping CI = 0.015, 0.159) in Table 3 indicates that the mediated relationship between family support and subjective well-being through work engagement was significantly moderated by religious attendance. The conditional indirect effect of family support on subjective well-being was higher among employees who had experience of religious attendance (B = 0.204, bootstrapping CI = 0.142, 0.273) than those who did not (B = 0.121, bootstrapping CI = 0.074, 0.169). The results thus support Hypothesis 2.

## 4. Discussion

The results support the mediating relationship between family support, work engagement, and subjective well-being as well as the moderating effect of religious attendance on the mediated relationship. That is, employee family support, work engagement, and subjective well-being exhibited a significant mediated relationship, and such a relationship strengthened when employees had religious attendance experience. The primary contribution of this study is as follows.

### 4.1. Emphasizing the Driving Effect of Family Support on High-Technology Employee Well-Being

Relying on the JD-R model and the positive affective spillover effect, this study revealed that family support exerted a critical driving effect on sense of well-being, particularly for high-technology employees working under a high level of job demand. Because family support is given without expecting anything in return, employees feel at ease while enjoying the love and care of family. Therefore, the positive influence of family support generates positive affects for employees in terms of work engagement, and they experience subjective well-being through the positive effects spilling over into their personal life.

### 4.2. Emphasizing the Moderating Effect of Religious Attendance as a Situational Strength

This study investigated the effect of a situational strength on the mediated relationship and was conducted in Taiwan to understand the effect of religion on high-technology employee well-being. The results indicate that the level of religious attendance experience moderated the relationship between family support, work engagement, and subjective well-being. Participating in religious activities allows employees who feel stress and pain from job demand to relieve the effects of the stress on their well-being. Moreover, employees with religious attendance experience tend to face stress with an optimistic attitude, which enables them to actively seek affective support from their social network to increase their sense of well-being and to reduce work-related stress [31,74]. Therefore, employees whose time at home is reduced by working overtime are advised to increase their time with family by attending religious activities with the entire family. Alternatively, immersion in religious doctrines can improve interaction between family members, thus giving employees family support [58]. Both serve to improve mental health as well as well-being at work and in one’s personal life.

## 5. Theoretical Implications

This study applied the JD-R model to explain the mechanism of job resources in a highly stressful working environment. The findings suggest that employees under high job demand should acquire resources from family and religion, which can help them cope with work-related stress and enhance their work engagement as well as personal well-being. However, the usefulness and ease of use of social media [75] have also enabled employees to relieve stress and obtain job resources, thus having a positive effect on employee well-being (Hoffman and Novak, 2012) [76]. Future researchers are advised to explore the mechanisms of social media in this regard and their relationship with work engagement and subjective well-being.

Owing to the COVID-19 pandemic, when employees experienced the threat of coronavirus disease in the workplace, working from home has been widely adopted as a flexible work arrangement [77]. In the work/home role transition, employees may prefer a work–home integration that brings their role at work to family life, or a work–home segmentation that separates their work role from their role at home [78]. The difference in preference can result in different work–home demarcation and influence work–home conflict in different ways [79], which may, in turn, affect work engagement and well-being. Therefore, future studies should also investigate how employee attitudes toward the work/home role transition potentially moderates the aforementioned mediated relationship.

## 6. Practical Implications

This study stressed the influence of family support and religious attendance on work engagement and subjective well-being. Organizations may provide employee-oriented flexible working arrangements [80] for employees who need to participate in religious activities or balance family obligations. Employees who have more time to devote to religious activities can obtain more family support and prevent work–family conflict [80], which can in turn help improve their work engagement and well-being. Organizations can also benefit from this, because happier employees exhibit greater performance and are more productive.

## 7. Limitations

This study used a cross-sectional research design, which involved employees responding to a questionnaire simultaneously. Future studies may consider collecting data at different time points. Also, self-reporting was used to measure the variables. To prevent biased responses pursuant to social desirability, all data were collected anonymously and used confidentially for academic purposes only. Future research may recruit respondents such as family members, coworkers, or supervisors as other sources reporting employee well-being both within and outside the workplace. Moreover, only 27% of the participants reported religious attendance outside work. However, people who do not attend religious activities may still be religious and understand religious doctrines and may be spiritual. Future studies might consider the individual’s overall relationship and diverse aspects toward religion and spirituality.

## 8. Conclusions

This study demonstrated the importance of well-being to employee innovative behavior, which is critical to the high-technology industry, by discussing and examining the relationship between family support, religious attendance, work engagement, and subjective well-being. This study focused on employees in high-technology industries, who, in the face of onerous job demands, may enhance subjective well-being through favorable resources outside the workplace. Family and religion are crucial domains in everyday life from which employees acquire personal resources. Compared with social support in the workplace, family support is less likely to create psychological burden on employees. Furthermore, this study emphasized the situational strength brought by religious attendance. Religious activities in Taiwan are temple activities [33], church activities, or rituals [34], and people typically follow their family members in praying for health and good luck. Participation in religious activities is also an opportunity for employees to spend time with their family members, and religious doctrines tend to prescribe strong familial relationships. We recommend paying attention to employee well-being because doing so is beneficial to employee quality of life and helps organizations to repeatedly gain product innovation and achieve sustainable development. Because this study was based on data collected in Taiwan alone, future studies should use data from other regions to verify the relationship between the variables assessed in this study. Finally, our study was conducted in the high-technology industry in Taiwan. Employees in the service industry may also suffer from emotional stress through interacting with customers, which may reduce well-being [81]. Future research can expand our understanding by applying our research to the service industry.

## Figures and Tables

**Table 1:** Descriptive Statistical Analysis and Correlation Analysis.

Variable	Mean	SD	1	2	3	4	5	6	7
1. Age	33.360	9.507	—						
2. Gender	0.577	0.494	0.215 **	—					
3. Tenure	6.810	6.900	0.772 **	0.229 **	—				
4. Family support	4.027	0.672	0.069	0.010	0.038	—			
5. Work engagement	3.348	0.655	0.149 **	−0.040	0.055	0.368 **	—		
6. Subjective well-being	2.240	0.528	0.075	−0.048	0.047	0.291 **	0.549 **	—	
7. Religious attendance	0.272	0.445	0.163 **	0.108 **	0.125 **	0.087 *	0.099 *	0.114 **	—

Note. Listwise *n* = 603. * *p* < 0.05, ** *p* < 0.01. For gender, 0 = male, 1 = female. For religious attendance, 0 = no, 1 = yes. SD: Standard deviation.

**Table 2:** Results of Mediation Analysis.

Variables	Work Engagement (M)				Subjective Well-Being (Y)			
	Coeff.	SE	LLCI	ULCI	Coeff.	SE	LLCI	ULCI
Work engagement (M)					0.414 ***	0.030	0.355	0.473
Family support (X)	0.348 ***	0.037	0.276	0.420	0.082 **	0.029	0.025	0.138
Age (U_1_)	0.016 ***	0.004	0.008	0.024	−0.003	0.003	−0.009	0.003
Gender (U_2_)	−0.086	0.051	−0.187	0.014	−0.035	0.037	−0.108	0.039
Tenure (U_3_)	−0.012 *	0.006	−0.023	−0.001	0.004	0.004	−0.004	0.013
Constant	1.539 ***	0.180	1.187	1.892	0.601 ***	0.139	0.328	0.874
*R* ^2^	0.162 ***				0.313 ***			
Indirect effects of X on Y	Effect		Boot SE		Boot LLCI		Boot ULCI	
X → M → Y	0.144		0.022		0.101		0.187	

Note. *n* = 603 (religious attendance, *n* = 164, no religious attendance, *n* = 439). 95% confidence interval. LLCI: lower-level confidence interval, ULCI: upper-level confidence interval. 5000 bootstrap resamples. * *p* < 0.05, ** *p* < 0.01, *** *p* < 0.001. SE: Standard error. →: The direction of causal relationship.

**Table 3:** Results of Moderated Mediation Analysis.

Variables	Work Engagement (M)				Subjective Well-Being (Y)			
	Coeff.	SE	LLCI	ULCI	Coeff.	SE	LLCI	ULCI
Work engagement (M)					0.414 ***	0.030	0.355	0.473
Family support (X)	0.293 ***	0.042	0.209	0.376	0.082 **	0.029	0.025	0.138
Religious attendance (W)	−0.740 *	0.346	−1.420	−0.060				
X × W	0.201 *	0.084	0.036	0.365				
Age (U_1_)	0.016 ***	0.004	0.008	0.024	−0.003	0.003	−0.009	0.003
Gender (U_2_)	−0.093	0.051	−0.193	0.007	−0.035	0.037	−0.108	0.039
Tenure (U_3_)	−0.012 *	0.006	−0.023	−0.001	0.004	0.004	−0.004	0.013
Constant	1.758 ***	0.198	1.369	2.147	0.601 ***	0.139	0.328	0.874
*R* ^2^	0.173 ***				0.313 ***			
Conditional indirect effects of X on Y	Effect		Boot SE		Boot LLCI		Boot ULCI	
Religious attendance (No)	0.121		0.024		0.074		0.169	
Religious attendance (Yes)	0.204		0.034		0.142		0.273	
Index of moderated mediation	Index		Boot SE		Boot LLCI		Boot ULCI	
X → M → Y by W	0.083		0.037		0.015		0.159	

Note. *n* = 603 (religious attendance, *n* = 164, no religious attendance, *n* = 439). 95% confidence interval. LLCI: lower-level confidence interval, ULCI: upper-level confidence interval. 5000 bootstrap resamples. * *p* < 0.05, ** *p* < 0.01, *** *p* < 0.001. →: The direction of causal relationship.

## Data Availability

The data underlying this research will be shared upon reasonable request to the corresponding author.

## References

[1] Khoreva V., Wechtler H. (2018). HR practices and employee performance: The mediating role of well-being. Empl. Relat..

[2] Ding H., Yu E. (2020). Follower strengths-based leadership and follower innovative behavior: The roles of core self-evaluations and psychological well-being. J. Work Organ. Psychol..

[3] Wright T.A., Cropanzano R. (2000). Psychological well-being and job satisfaction as predictors of job performance. J. Occup. Health.

[4] Zelenski J.M., Murphy S.A., Jenkins D.A. (2008). The Happy-Productive Worker Thesis Revisited. J. Happiness Stud..

[5] Zhang X., Lin Z., Liu Y., Chen X., Liu D.M. (2020). How do human resource management practices affect employee well-being? A mediated moderation model. Empl. Relat..

[6] Schaufeli W.B., Salanova M., González-Romá V., Bakker A.B. (2002). The measurement of engagement and burnout: A two sample confirmatory factor analytic approach. J. Happiness Stud..

[7] Xanthopoulou D., Bakker A.B., Demerouti E., Schaufeli W.B. (2009). Reciprocal relationships between job resources, personal resources and work engagement. J Vocat. Behav..

[8] Tims M., Bakker A.B., Derks D. (2013). The impact of job crafting on job demands, job resources, and well-being. J. Occup. Health Psychol..

[9] Schnettler B., Denegri M., Miranda H., Sepu’lveda J., Orellana L., Paiva G., Grunert K.G. (2015). Family support and subjective well-being: An exploratory of university students in southern chile. Soc. Indic. Res..

[10] Toplu-Demirtas E., Kemer G., Pope A.L., Moe J.L. (2018). Self-compassion matters: The relationships between perceived social support, self-compassion, and subjective well-being among LGB individuals in Turkey. J. Couns. Psychol..

[11] Culbertson S.S., Mills M.J., Fullagar C.J. (2012). Work engagement and work-family facilitation: Making Homes happier through positive affective spillover. Hum. Relat..

[12] Matthews R.A., Mills M., Trout R.C., English L. (2014). Family-supportive supervisor behaviors, work engagement, and subjective well-being: A contextually dependent mediated process. J. Occup. Health Psychol..

[13] Bakker A.B., Demerouti E., Verbeke W. (2004). Using the job demands-resources model to predict brunout and performance. Hum. Resour. Manag..

[14] Cheung C.M., Zhang R.P., Cui Q., Hsu S.-C. (2021). The antecedents of safety leadership: The job demands-resources model. Saf. Sci..

[15] Hara Y., Asakura K., Sugiyama S., Takada N., Ito Y., Nihei Y. (2021). Nurses working in nursing homes: A mediation model for work engagement based on job demands-resources theory. Healthcare.

[16] Park J.H., Chang Y.K., Kim S. (2021). Are your vitals ok? Revitalizing vitality of nurses through relational caring for patients. Healthcare.

[17] D’Aveni R.A., Dagnino G.B., Smith K.G. (2010). The age of temporary advantage. Strateg. Manag. J..

[18] Martín-Hernández P., Ramos J., Zornoza A., Lira E.M., Peiró J.M. (2020). Mindfulness and job control as moderators of the relationship between demands and innovative work behaviours. J. Work Organ. Psychol..

[19] Döckel A., Basson J., Coetzee M. (2006). The effect of retention factors on organizational commitment: An investigation of high technology employees. J. Hum. Resour. Manag..

[20] Chernyak-Hai L., Tziner A. (2016). The “I believe” and the “I invest” of work-family balance: The indirect indirect influences of personal values and work engagement via perceived organizational climate and workplace burnout. J. Work Organ. Psychol..

[21] Bryant R.M., Constantine M.G. (2006). Multiple role balance, job satisfaction, and life satisfaction in woman school counselors. Prof. Sch. Couns..

[22] Snir R., Harpaz I., Ben-Baruch D. (2009). Centrality of and investment in work and family among Israeli high-tech workers: A bicultural perspective. Cross Cult. Res..

[23] Shin Y., Hur W.-M., Choi W.-H. (2020). Coworker support as a double-edged sword: A moderated mediation model of job crafting, work engagement, and job performance. Int. J. Hum. Resour. Manag..

[24] Tews M.J., Michel J.W., Ellingson J.E. (2013). The impact of coworker support on employee turnover in the hospitality industry. Group Organ. Manag..

[25] Poortvliet P.M., Janssen O., Van Yperen N.W., Van de Vliert E. (2009). Low ranks make the difference: How achievement goals and ranking information affect cooperation intentions. J. Exp. Soc. Psychol..

[26] Murphy S.M., Wayne S.J., Liden R.C., Erdogan B. (2003). Understanding social loafing: The role of justice perceptions and exchange relationships. Hum Relat..

[27] Greenhaus J.H., Powell G.N. (2006). When work and family are allies: A theory of work-family enrichment. Acad. Manag. Rev..

[28] Prazeres F., Passos L., Simões J.A., Simões P., Martins C., Teixeira A. (2021). COVID-19-Related fear and anxiety: Spiritual-religious coping in healthcare workers in Portugal. Int. J. Environ. Res. Public Health.

[29] Underwood L.G. (2000). A working model of health: Spirituality and religiousness as resources: Applications to persons with disability. J. Relig. Disabil. Health.

[30] Cash K.C., Gary G.R. (2000). A framework for accommodating religion and spirituality in the workplace. Acad. Manag. Perspect..

[31] Ellison C.G. (1991). Religious involvement and subjective Well-Being. J. Health Soc. Behav..

[32] Acevedo G.A., Ellison C.G., Xu X. (2014). Is it really religion? Comparing the main and stress-buffering effects of religious and secular civic engagement on psychological distress. Soc. Ment. Health.

[33] Chang W. (2009). Religious attendance and subjective well-being in an eastern-culture country: Empirical evidence from Taiwan. Marbgurg J. Relig..

[34] Cappellen P.V., Toth-Gauthier M., Saroglou V., Fredrickson B.L. (2016). Religion and well-being: The mediating role of positve emotions. J. Happiness Stud..

[35] Wilcox W.B., Chaves M., Franz D. (2004). Focused on the family? Religious traditions, family discourse, and pastoral practice. J. Sci. Stud. Relig..

[36] Johns G. (2006). The essntial impact of context on organizational behavior. Acad. Manag. Rev..

[37] Bakker A.B., Hakanen J.J., Demerouti E., Xanthopoulou D. (2007). Job resources boost work engagement particularly when job demands are high. J. Educ. Psychol..

[38] Bakker A.B., Demerouti E. (2008). Towards a model of work engagement. Career Dev. Int..

[39] Näwall K., Sverke M., Göransson S. (2014). Is work affecting my health? Appraisals of how work affects health as a mediator in the relationship between working conditions and work-related attitudes. Work Stress.

[40] Ahmed I., Islam T., Usman A. (2020). Predicting entrepreneurial intentions through self-efficacy, family support, and regret: A moderated mediation explanation. J. Entrep. Emerg. Econ..

[41] Leung Y.K., Mukerjee J., Thurik R. (2020). The role of family support in work-family balance and subjective well-being of SME owners. J. Small Bus. Manag..

[42] Decuypere A., Schaufeli W. (2020). Leadership and work engagement: Exploring explanatory mechanisms. Ger. J. Hum. Resour. Manag..

[43] Schaufeli W.B., Bakker A.B. (2004). Job demands, job resources, and their relationship with burnout and engagement: A multi-sample study. J. Organ. Behav..

[44] Chen I.-S., Fellenz M.R. (2020). Personal resources and personal demands for work engagement: Evidence from employees in the service industry. Int. J. Hosp. Manag..

[45] Diener E., Lucas R.E., Oishi S. (2002). Subjective Well-Being: The Science of Happiness and Life Satisfaction.

[46] Diener E. (1984). Subjective well-being. Psychol. Bull..

[47] Darvishmotevali M., Ali F. (2020). Job insecurity, subjective well-being and job performance: The moderating role of psychological capital. Int. J. Hosp. Manag..

[48] Sonnentag S. (2003). Recovery, work engagement, and proactive behavior: A new look at the interface between nonwork and work. J. Appl. Psychol..

[49] Wayne J.H., Randel A.E., Stevens J. (2006). The role of identity and work-family support in work-family enrichment and its work-related consequences. J. Vocat. Behav..

[50] William K.J., Alliger G.M. (1994). Role stressors, mood spillover, and perceptions of work-family conflict in employed parents. Acad. Manag. J..

[51] Liao K.Y., Weng C. (2018). Gratefulness and subjective well-being: Social connectedness and presence of meaning as mediators. J. Couns. Psychol..

[52] Yu S., Levesque-Bristol C., Maeda Y. (2018). General need for autonomy and subjective well-being: A meta-analysis of studies in the US and East Asia. J. Happiness Stud..

[53] Lu L. (2006). “Culture fit”: Individual and societal discrepancies in values, beliefs, and subjective well-being. J. Soc. Psychol..

[54] Siedlecki K.L., Salthouse T.A., Oishi S., Jeswani S. (2014). The relationship between social support and subjective well-being accross age. Soc. Indic. Res..

[55] Rusu P.P., Colomeischi A.A. (2020). Positivity ratio and well-being among teachers. The mediating role of work engagement. Front. Psychol..

[56] Levin J.S., Markides K. (1988). Religious attendance and psychological well-being in middle-aged and older Mexican Americans. Sociol. Anal..

[57] George L.K., Ellison C.G., Larson D.B. (2002). Explaining the relationship between religious involvement and health. Psychol. Inq..

[58] Ellison C.G., Burdette A.M., Wilcox W.B. (2010). The couple that prays together: Race, ethnicity, religion, and relationship quality among working-age adults. J. Marriage Fam..

[59] Ryan R.M., Deci E.L. (2000). Self-determination theory and the facilitation of intrinsic motivation, social development, and well-being. Am. Psychol..

[60] Wong J.-Y., Lin J.-H., Liu S.-H. (2008). Coping with work-nonwork conflict and promoting life quality of frontline employees via social support. J. Manag. Syst..

[61] King L.A., Mattimore L.K., King D.W., Adams G.A. (1995). Family support inventory for workers: A new measure of perceived social support from family member. J. Organ. Behav..

[62] Lien C.-L. (2012). The relationships between presidents’ authentic leadership and teachers’ organizational commitment in higher education: The mediation effect of teachers’ work engagement. Serv. Ind. Manag. Rev..

[63] Lu L., Hwang M.-T., Kao S.-F. (2005). The Bi-directional conflict of work and family: Antecedents, consequences and moderators. Res. Appl. Psychol..

[64] Lu L., Gilmour R., Kao S., Huang M. (2006). A cross-cultural study of work/family demands, work/family conflict and wellbeing: The Taiwanese vs. British. Career Dev. Int..

[65] Presser S., Stinson L. (1996). Estimating the bias in survey reports of religious attendance. Survey Research Methods.

[66] Presser S., Stinson L. (1998). Data collection mode and social desirability bias in self-reported religious attendance. Am. Sociol. Rev..

[67] Acuña E., Rodriguez C. (2004). The treatment of missing values and its effect in the classifier accuracy. Classification, Clustering and Data Mining Applications.

[68] Hayes A.F. (2018). Introduction to Mediation, Moderation, and Conditional Process Analysis: A Regression-Based Approach.

[69] Rothbard N.P. (2001). Enriching or Depleting? The dynamics of engagement in work and family roles. Adm. Sci. Q..

[70] Avolio B., Yammarino F.J., Bass B.M. (1991). Identifying common methods variance with data colleted from a single source: An unresolved sticky issue. J. Manag..

[71] Schmitt N. (1994). Method Bias: The importance of theory and measurement. J. Organ. Behav..

[72] Podsakoff P.M., Organ D.W. (1986). Self-reports in orgaizational research: Problems and prospects. J. Manag..

[73] Fuller C.M., Simmering M.J., Atinc G., Atinc Y., Babin B.J. (2016). Common methods variance detection in business research. J. Bus. Res..

[74] Nooney J.G. (2005). Stress, and mental health in adolescence: Findings from Add Health. Rev. Relig. Res..

[75] Rauniar R., Rawski G., Yang J., Johnson B. (2014). Technology acceptance model (TAM) and social media usage: An empirical study on Facebook. J. Enterp. Inf. Manag..

[76] Hoffman D.L., Novak T. Why do People Use Social Media? Empirical Findings and a New Theoretical Framework for Social Media Goal Pursuit. https://papers.ssrn.com/sol3/papers.cfm?abstract_id=1989586.

[77] Avgoustaki A., Bessa I. (2019). Examining the link between flexible working arrangement bundles and employee work effort. Hum. Res. Manag..

[78] Ashforth B.E., Kreiner G.E., Fugate M. (2000). All in a day’s work: Boundaries and micro role transitions. Acad. Manag. Rev..

[79] Kreiner G.E. (2006). Consequences of work-home segmentation or integration: A person-environment fit perspective. J. Organ. Behav..

[80] Allen T.D., Johnson R.C., Kiburz K.M., Shockley K.M. (2013). Work–family conflict and flexible work arrangements: Deconstructing flexibility. Pers. Psychol..

[81] Pugh S.D., Groth M., Hennig-Thurau T. (2011). Willing and able to fake emotions: A closer examination of the link between emotional dissonance and employee well-Being. J. Appl. Psychol..

